# The Role of Cytokinins and Abscisic Acid in the Growth, Development and Virulence of the Pathogenic Fungus *Stagonospora nodorum* (Berk.)

**DOI:** 10.3390/biom14050517

**Published:** 2024-04-25

**Authors:** Tatyana V. Nuzhnaya, Antonina V. Sorokan, Guzel F. Burkhanova, Igor V. Maksimov, Svetlana V. Veselova

**Affiliations:** 1 Institute of Biochemistry and Genetics, Ufa Federal Research Centre, Russian Academy of Sciences, Prospekt Oktyabrya, 71, 450054 Ufa, Russia; tanyawww89@mail.ru (T.V.N.); fourtyanns@googlemail.com (A.V.S.); guzel_mur@mail.ru (G.F.B.); maksimov@ufaras.ru (I.V.M.); 2 Ufa Institute of Biology, Ufa Federal Research Centre, Russian Academy of Sciences, Prospekt Oktyabrya, 69, 450054 Ufa, Russia

**Keywords:** carbohydrate metabolism, fungal development, hormones, necrotrophic effectors (NEs), plant growth regulators (PGRs), sporulation, transcription factors (TFs)

## Abstract

Cytokinins (CKs) and abscisic acid (ABA) play an important role in the life of both plants and pathogenic fungi. However, the role of CKs and ABA in the regulation of fungal growth, development and virulence has not been sufficiently studied. We compared the ability of two virulent isolates (SnB and Sn9MN-3A) and one avirulent isolate (Sn4VD) of the pathogenic fungus *Stagonospora nodorum* Berk. to synthesize three groups of hormones (CKs, ABA and auxins) and studied the effect of exogenous ABA and zeatin on the growth, sporulation and gene expression of necrotrophic effectors (NEs) and transcription factors (TFs) in them. Various isolates of *S. nodorum* synthesized different amounts of CKs, ABA and indoleacetic acid. Using exogenous ABA and zeatin, we proved that the effect of these hormones on the growth and sporulation of *S. nodorum* isolates can be opposite, depends on both the genotype of the isolate and on the concentration of the hormone and is carried out through the regulation of carbohydrate metabolism. ABA and zeatin regulated the expression of fungal TF and NE genes, but correlation analysis of these parameters showed that this effect depended on the genotype of the isolate. This study will contribute to our understanding of the role of the hormones ABA and CKs in the biology of the fungal pathogen *S. nodorum*.

## 1. Introduction

Phytohormones are the alpha and omega of regulation of the growth, development, reproduction and, ultimately, total metabolism of plants [1,2,3,4]. In addition, phytohormones’ auxins, cytokinins (CKs), gibberellins, ethylene, abscisic acid (ABA), jasmonic acid and salicylic acid induce and coordinate signaling pathways involved in various plant responses to biotic and abiotic environmental stress factors [1,3,4]. However, most classical plant hormones are also produced by pathogenic and symbiotic fungi [5,6,7]. Today, it is believed that hormones synthesized by fungi have two vectors of action. The first vector is aimed at regulating interactions with plants and other organisms. In this case, hormones play the role of effectors and can determine the rate of virulence of the fungus. As effectors, hormones regulate the penetration of the pathogen into the plant tissues and the absorption of nutrients by fungal mycelium [5]. The second vector is aimed at regulating the processes of growth and development of the fungi themselves, as well as their adaptation to environmental conditions [5]. Unfortunately, the participation of hormonal compounds, which are synthesized by microorganisms in the development of fungi and in the interactions of plants and pathogenic fungi, has practically not been studied.

Currently, it is believed that CKs and ABA play an important role in regulating the interaction between plants and pathogens [1,2,3,4]. However, information about the role of these hormones in the development of plant defense reactions is contradictory, and evidence of the role of CKs and ABA in the regulation of fungal growth and development is very limited [5,8].

CKs are relatively simple derivatives of adenine or adenosine, modified at the nitrogen atom in the sixth position of a six-membered heterocycle [9]. Little is known about the functions of CKs in fungi, but some studies show the influence of CKs on fungal development, hyphal growth, nutrient absorption and ion and water transport [5,10,11]. However, recent work showed that CKs strongly inhibited the growth of *Botrytis cinerea*, attenuating the cell cycle and reducing cytoskeletal organization [10]. Thus, the direct effect of CKs on phytopathogens is of great interest, and the available data are very contradictory, so this issue requires further study.

Sesquiterpene ABA is known as a plant hormone that regulates stress tolerance. However, ABA biosynthesis is found in a phylogenetically wide range of organisms [12], from cyanobacteria, fungi and sponges to human cells [13]. Recent studies have shown that ABA can directly influence the growth and development of fungi, increase the germination of spores and the formation of appressoria, control the flow of water and mineral salts, regulate carbohydrate metabolism and sugar transport and also increase the virulence of pathogens [14,15,16,17]. For these reasons, it is very important to understand the mechanism of action of ABA in non-plant organisms and to elucidate the biological significance of ABA as a “universal signaling molecule” [15].

Fungal diseases annually lead to significant crop losses of the most important food culture *Triticum aestivum* L. [18]. Septoria nodorum blotch (SNB), caused by the pathogenic fungus *Stagonospora nodorum* (Berk.), affects wheat leaves and ears [18]. *S. nodorum* belongs to the order Pleosporales of the class Dothideomycetes (teleomorph *Phaeosphaeria nodorum*, *Parastagonospora nodorum*). It is one of the most harmful necrotrophic pathogens of wheat, which can infect all above-ground parts of plants, and it is widespread in wheat-growing regions, including North America, Australia, Europe and Asia, where it can cause serious economic damage [19,20].

Despite the importance of *S. nodorum* among wheat diseases, the spectrum of secondary metabolites (SMs) of this fungus has not been well studied. Nothing is known about the synthesis and secretion of hormonal compounds by *S. nodorum*. It is known that *S. nodorum* produces low-molecular-weight SMs, such as septorines, melleins and mycophenolic acids [18]. In addition, *S. nodorum* produces protein necrotrophic effectors (NEs) as the major virulence factor [20]. NEs ensure the virulence of *S. nodorum* strains in the host plant, in the genome of which there is a dominant susceptibility gene corresponding to the effector [20].

To date, NEs of *S. nodorum* SnToxA, SnTox1, SnTox267, SnTox3, SnTox4 and SnTox5 have been identified and characterized to some extent [21,22,23,24,25]. Gene and protein sequences of SnToxA, SnTox1 and SnTox3 have been identified, and these NEs are considered the most common among strains and isolates of the pathogen [26,27]. Despite the fact that the main function of NEs of the pathogen *S. nodorum* is induction of host cell death by manipulating plant defense signaling pathways, including hormonal cross-talk, in many cases, the role of NEs remains unclear.

It is obvious that studying the mechanisms of the regulation of the expression of effector genes, which are key determinants of fungal pathogenicity, will open up opportunities for the development of new plant protection strategies. However, mechanisms of the expression of NE genes of *S. nodorum* are multidimensional, and knowledge of how NE genes are regulated is still largely lacking. Recent advances in sequencing and annotation of fungal genomes have revealed a variety of fungal transcription factors (TFs) that play a certain role in the regulation of many processes in the pathogen, such as carbohydrate metabolism, hyphal growth, enzyme biosynthesis and sporulation [28]. TFs also influence the virulence of the pathogen and regulate the expression of NE genes [28]. Thus, in the pathogen *S. nodorum*, TFs *SnPf2*, *SnStuA* and *SnCon7*, which are capable of regulating the expression of NE genes, were discovered [28,29,30,31]. Along with TFs, the growth, development and virulence of pathogens are regulated by hormones of fungal origin, which play an important role in the life of fungi [5]. However, the role of fungal-derived hormones in the regulation of pathogen virulence and the expression of NE and TF genes has been practically unstudied. We hypothesize that not only NEs can manipulate hormonal signaling pathways, but hormones can also regulate the development and pathogenicity of the fungus by influencing NEs and TFs.

Previously, we showed that isolates of *S. nodorum* SnB and Sn9MN-3A contain *SnToxA* and *SnTox3* in their genome sequences of NEs, and these isolates are virulent against wheat genotypes carrying the corresponding susceptibility genes complementary to these effectors [32,33]. The *S. nodorum* Sn4VD isolate contained in its genome the genes encoding three NEs: *SnTox1*, *SnToxA* and *SnTox3*, but the Sn4VD isolate was avirulent and weakly infected susceptible wheat genotypes [32].

In this regard, the purpose of our research was to study the effect of a CK (zeatin) and ABA on the growth of fungal colonies, the degree of sporulation and the expression of genes encoding a number of different virulence factors in various isolates of the pathogen *S. nodorum*. To identify the role of hormones in the regulation of sporulation of the fungus *S. nodorum*, the expression of mannitol (*Mdh1* and *Mpd1*) and trehalose (*Tps1*) biosynthesis genes was studied in different isolates of the pathogen growing on a medium containing zeatin or ABA. To identify the influence of zeatin and ABA on the virulence factors of *S. nodorum* isolates, the expression of genes encoding NEs (*SnToxA*, *SnTox1* and *SnTox3*) and TFs (*SnStuA*, *SnPf2* and *SnCon7*) of different isolates of the pathogen (SnB, Sn9MN-3A and Sn4VD) growing on a medium with the addition of zeatin or ABA was studied. Correlation matrices were constructed that showed the multidirectional influence of zeatin and ABA on the virulence factors of the pathogen *S. nodorum*, depending, among other things, on the genotype of the isolates. These results will contribute to the understanding of the role of the CKs and ABA in the processes of pathogen growth and development, as well as in the regulation of *S. nodorum* NE and TF expression.

## 2. Materials and Methods

### 2.1. Fungi Material and Growth Conditions

Isolates of the fungus *S. nodorum*, Sn4VD, SnB and Sn9MN-3A (from the collection of the Institute of Biochemistry and Genetics, Ufa Federal Research Center of the Russian Academy of Sciences, Ufa, Russia, https://ibg.anrb.ru/naychnaya-deyatelnost/bioresyrs/kollekciya-simbioticheskix-mikroorganizmov/ (accessed on 25 October 2023), were investigated.

Solid potato glucose agar (PGA) supplemented with glucose 20 g∙L^−1^ was used to study growth characteristics and sporulation. Liquid potato dextrose broth (PGB) with glucose 20 g∙L^−1^ was used to study the synthesis of hormones in *S. nodorum* and the expression of fungal genes.

*S. nodorum* isolates were maintained at 4 °C on barley grains. To obtain the fungal spore preparation, barley grains containing the sporomycelial mass were soaked in sterile distilled water (distiller A1110, LLC “Liston”, Zhukov, Russia), then the spore suspension was applied to the surface of PGA with glucose 20 g∙L^−1^ and kept in a thermostat (TC 1/20 SPU, Smolenskoye SKTB SPU, Smolensk, Russia) at 18 °C for 14 days. Sections (5 × 5 mm) of mycelium (or mycelium with spores) from the 14-day-old colonies were used for further experiments, which were transferred to PGA or PGB with or without the addition of plant growth regulators (PGRs) ABA or zeatin. Initially, zeatin (Merck KGaA, Sigma-Aldrich, Darmstadt, Germany) and ABA (Merck KGaA, Sigma-Aldrich, Darmstadt, Germany) were dissolved in a drop of 96% ethanol and then diluted in distilled water to a final concentration of 1 mg∙mL^−1^. Stock solutions were filter-sterilized (0.22 μm filter, Merck KGaA, Sigma-Aldrich, Darmstadt, Germany) and added to the PGA or PGB medium after autoclaving and the cooldown of the medium at final concentrations from 0.001 to 0.1 µM (from 0.2 to 22 ng∙mL^−1^ of zeatin, from 0.3 to 26.4 ng∙mL^−1^ of ABA). The studies were carried out under aseptic conditions using a laminar flow safety cabinet BAVp-01 (Lamsystems, Miass, Russia). Selection of the concentration of PGRs that have the greatest effect on fungal sporulation was carried out with the SnB isolate. In further investigations of *S. nodorum* isolates carrying different sets of effector genes and TF genes, we used 0.1 μM PGRs (zeatin and ABA).

### 2.2. Assay of the Fungal-Derived Hormone Content in the Mycelium and Liquid Culture Medium of S. nodorum

The liquid culture filtrate and mycelium obtained by growing *S. nodorum* isolates Sn4VD, SnB and Sn9MN-3A for 21 days were used for hormone analysis. Also, to check the background level of hormones, pure culture medium (CM) was taken for analysis at the same time as fungal cultures. The mycelium (approximately 1 g from one flask) per one biological replication was homogenized, and hormones were extracted with 80% ethanol (1:10, weight/volume) for 16 h at 4 °C. The extracts from the mycelium were centrifuged at 4000× *g* for 20 min in an Avanti J-E centrifuge (Beckman Coulter, Bray, OK, USA) and evaporated to obtain aqueous residue. The culture medium was collected and centrifuged at 4000× *g* for 20 min in an Avanti J-E centrifuge (Beckman Coulter, Bray, OK, USA). The supernatant was analyzed for the content of hormones (cytokinins, indole-3-acetic acid (IAA) and ABA). Three independent biological replicates were performed for each experiment.

Cytokinins from 2 mL of the supernatant of the fungal liquid medium were twice extracted with n-butyl alcohol in a 2:1 ratio (aqueous phase/organic phase). The extract was evaporated to dryness. Cytokinins from the aqueous residue of the mycelium extract were concentrated on a C18 column (Waters Corporation, Milford, MA, USA), eluted with 5 mL of 80% ethanol and then evaporated to dryness. Cytokinin bases and their derivatives from dry residues in both cases were separated by thin layer chromatography on Silufol plates (Merck KGaA, Fluka, Darmstadt, Germany) in a system with the solvents butanol/ammonium hydrate/water (6:1:2), according to [34]. In this work, we analyzed the riboside of zeatin (ZR, Rf 0.4–0.5) and zeatin (Z, Rf 0.6–0.7). The material from different zones was eluted, and afterward, it was assayed by enzyme-linked immunosorbent assay (ELISA) using specific antibodies, as described earlier [34].

IAA and ABA from 1 mL of the supernatant of the fungal liquid culture and from 1 mL of the aqueous residue of the mycelium extract were extracted with diethyl ether, according to a modified scheme [35]. The IAA and ABA quantitative assay was performed with ELISA using specific antibodies, as described previously [35]. The reliability of the hormone immunoassay was confirmed using a dilution test and through a comparison of the data obtained with the results of high-performance liquid chromatography (HPLC) in combination with mass spectrometry [34,36].

### 2.3. Assay of Growth Characteristics and Sporulation in S. nodorum Isolates

The diameter of the colonies and the mycelium square were measured on the 1st, 2nd, 3rd, 5th, 7th and 14th days of growth. The intensity of sporulation was assessed visually and by direct counting on the 2nd, 3rd, 5th, 7th and 14th days of growth. To count the number of spores, the surface of the colonies was filled with 5 mL of distilled water and incubated for 10 min; spores in aliquots were counted using a Fuchs-Rosenthal chamber [37]. The intensity of sporulation was measured by the number of spores per 1 cm^2^ of the colony. Mycelium images were obtained using a Scanjet G4050 scanner (HP Inc. (Palo-Alto, CA, USA) or an SP-800UZ Image Stabilization camera (Olympus, Tokyo, Japan).

The augmentation of spores (*ASp*) for each following period (*i*) was calculated using Equation (1):

(1) $${ASp}_{i}={Sp}_{i}-{Sp}_{i-x},$$

where *Sp_i_* is the number of spores formed per 1 cm^2^ of colony before the *i*-day of cultivation (*i* = 2, 3, 5, 7, 14) and *Sp_i−x_* is the number of spores formed per 1 cm^2^ of colony before the (*i − x*)-day of cultivation, where *x* is the difference between the *i*-day of measurement and the previous one. The mycelium growth rate (*Vm*) for each following period (*i*) was calculated using Equation (2):

(2) $${Vm}_{i}=\frac{\left(S_{i}-S_{i-x}\right)}{x}$$

where *S_i_* is the mycelium square before the *i*-day of cultivation (*i* = 1, 2, 3, 5, 7, 14) and *S_i−x_* is the mycelium area before the (*i − x*)-day of cultivation, where *x* is the difference between the *i*-day of measurement and the previous one.

### 2.4. Assay of Gene Expression

Mycelium of *S. nodorum* SnB, Sn4VD and Sn9MN-3A from three flasks per biological replication was collected and fixed in liquid nitrogen on the 2nd (mycelial growth before the start of sporulation or the very beginning of sporulation), the 5th (active phase of sporulation), the 7th (continued growth and sporulation) and the 14th (continued growth and sporulation) days of cultivation on PGB.

Total fungal RNA was extracted using Lira^®^ (Biolabmix, Moscow, Russia) according to the manufacturer’s instructions. cDNA synthesis was carried out as described previously [38]. Primers for real-time polymerase chain reaction (real-time PCR) were devised using the web tool PrimerQuest™ (https://www.idtdna.com/pages/tools/primerquest, accessed on 10 November 2023) (Integrated DNA Technologies, Inc., Coralville, IA, USA). The sequences of all the primers are presented in Table S1 (Supplementary Materials) for genes encoding NEs (*SnTox1*, *SnToxA* and *SnTox3*), TFs (*SnPf2*, *SnStuA* and *SnCon7*), enzymes of mannitol synthesis (mannitol 2-dehydrogenase (gene *Mdh1*) and mannitol-1-phosphate dehydrogenase (gene *Mpd1*)) and the gene encoding the enzyme of the trehalose synthesis (trehalose-6-phosphate synthase (gene *Tps1*)). Primers were selected so that their annealing temperature was 60 °C. A melting curve analysis was conducted to determine the specificity of the reaction (at 95 °C for 10 s, 65 °C for 5 s and 95 °C for 5 s). Real-time PCR was performed on a “DNA amplifier in real time” CFX96 (BioRad Laboratories, Hercules, CA, USA) using a set of reagents, Eva-Green Supermix (Syntol, Moscow, Russia), according to the manufacturer’s protocols. To standardize the data, fungal genes *Snβ*-*tubulin* and elongation factor 1α (*SnEF-1α*) (Table S1, Supplementary Materials) were used as an internal reference for the real-time PCR analysis. In order to quantify the relative gene expression, the delta-delta Ct method was performed, as described earlier [38]. Three independent biological and three technical replications were performed for each experiment.

### 2.5. Statistical Analysis

All experiments were repeated three times with three biological repetitions. Experimental data were expressed as means ± SE, which were calculated in all treatments using Microsoft Excel (version 16.0.14430.20306, Redmond, WA, USA). The significance of differences was assessed by ANOVA followed by Duncan’s test (*p* ≤ 0.05) with STATISTICA 10.0 software (version STA999K347150-W, Tulsa, OK, USA). Before statistical analysis, the normal distribution of all parameters was analyzed and confirmed by the Shapiro–Wilk W test. The calculation of Pearson’s correlation coefficients and the construction of correlation matrices were carried out using Microsoft Excel (version 16.0.14430.20306, Redmond, WA, USA). The treatment variants and the number of repetitions are indicated in the tables and figures.

## 3. Results

### 3.1. Ability of Different Isolates of S. nodorum to Produce Hormones

It was shown that *S. nodorum* isolates SnB, Sn9MN-3A and Sn4VD accumulated ABA, IAA or CKs mainly in the mycelium and secreted a small amount of the hormones into the culture medium (Table 1).

The SnB isolate synthesized ABA, IAA and trace amounts of CKs (the sum of zeatin and zeatin riboside) (Table 1). Trace amounts of ABA, IAA and CKs were detected in the pure culture medium (Table 1). The Sn9MN-3A isolate synthesized slightly less amount of ABA and the same amount of IAA as compared with the SnB isolate. The Sn9MN-3A isolate accumulated some CKs in the mycelium and also secreted hormones under consideration into the culture medium (Table 1). The Sn4VD isolate accumulated almost three times less ABA in the mycelium than the SnB isolate but synthesized two times more IAA and more than 100 times as many CKs as the SnB isolate (Table 1). The Sn4VD isolate secreted ABA in similar quantities as the SnB isolate into the culture medium, but IAA and CKs were secreted in much greater quantities than the SnB isolate (Table 1).

### 3.2. The Influence of Zeatin and ABA on the Growth and Sporulation of the Pathogen S. nodorum

#### 3.2.1. Selection of Concentrations of Zeatin and ABA Affecting the Growth and Sporulation of the Pathogen

**Figure 1:**
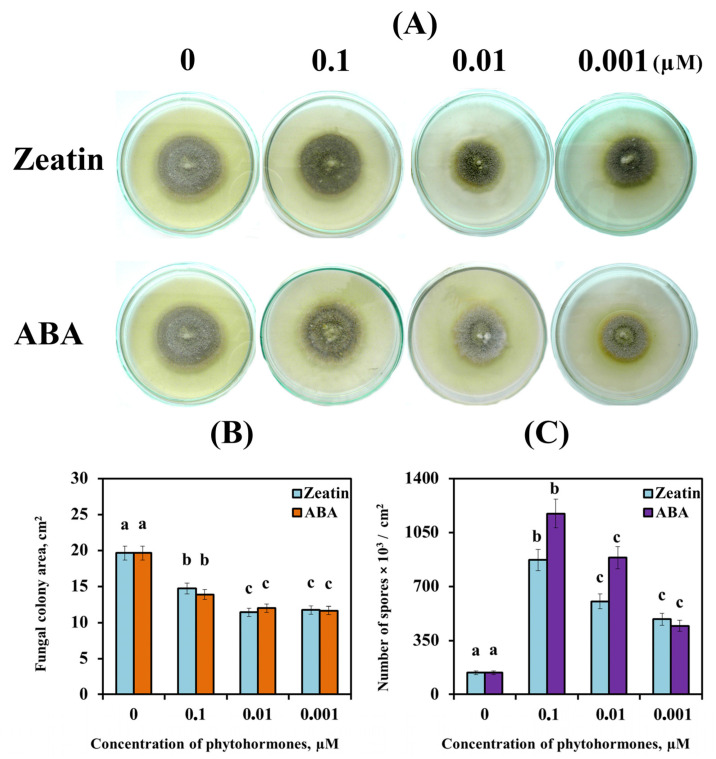
The influence of different concentrations of ABA and zeatin (from 0.001 to 0.1 µM) on the morphology (**A**), radial growth of the mycelium (**B**) and sporulation (**C**) of *S. nodorum* SnB isolate cultivated on PGA for 7 days (**A**) and 14 days (**B**,**C**). 0 µM—PGA medium without addition of ABA or zeatin. Figures present means ± SE (*n* = 9). The variants on each histogram marked with similar letters do not differ significantly according to Duncan’s test (*p* ≤ 0.05).

Our results showed that zeatin and ABA did not affect the growth of the SnB isolate mycelium (Figure 1A). However, PGRs (zeatin and ABA) approximately equally affected the growth of the fungal colony, reducing the area of mycelium (Figure 1B). Low concentrations of zeatin or ABA (0.001 and 0.01 µM) reduced the area of mycelium by 39–42% compared to the control plates without PGRs (Figure 1B). A higher concentration (0.1 μM) of zeatin or ABA affected growth to a lesser extent, reducing the area of mycelium by only 25 and 29%, respectively (Figure 1B).

The decrease in the area of the fungal colonies under the influence of zeatin or ABA was compensated by an increase in sporulation (Figure 1C). ABA increased fungal sporulation to a greater extent than zeatin, especially at higher concentrations (0.1 and 0.01 μM) (Figure 1C). Thus, the addition of 0.1 μM zeatin to the culture medium increased the sporulation of SnB isolate six times, and the same concentration of ABA increased fungal sporulation eight times (Figure 1C). With a decrease in the concentration of zeatin and ABA, their effect on the number of *S. nodorum* spores decreased; however, even at the lowest concentration of 0.001 μM, both PGRs increased sporulation by 3–3.5 times compared to control colonies (Figure 1B).

Thus, 0.1 μM of zeatin and ABA had the greatest effect on the sporulation of the fungus and the least inhibiting effect on the radial growth of the mycelium (Figure 1).

#### 3.2.2. Effect of Zeatin and ABA on Mycelial Growth and Sporulation of *S. nodorum* Isolates

**Figure 2:**
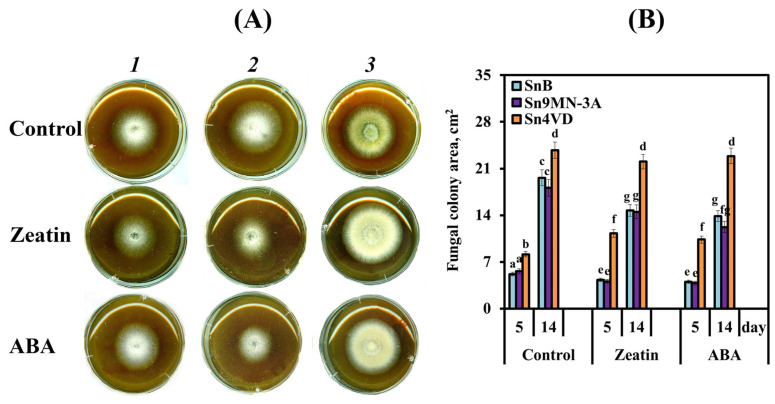
The influence of ABA (0.1 µM) and zeatin (0.1 µM) on the morphology (**A**) of *S. nodorum* isolates SnB (*1*), Sn9MN-3A (*2*) and Sn4VD (*3*) cultivated on PGA for 5 days and radial growth of the mycelium of these isolates (**B**), cultivated on PGA for 5 and 14 days. Control is a PGA medium without the addition of zeatin or ABA. Figures present means ± SE (*n* = 9). The variants on histogram marked with similar letters do not differ significantly according to Duncan’s test (*p* ≤ 0.05).

We compared the influence of ABA and zeatin on the radial growth, morphology and sporulation of *S. nodorum* isolates SnB, Sn9MN-3A and Sn4VD cultured on PGA (Figure 2A).

**Figure 3:**
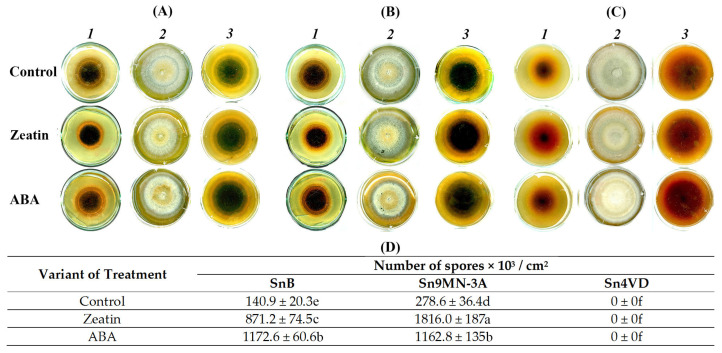
The effect of ABA (0.1 µM) and zeatin (0.1 µM) on the morphology of *S. nodorum* isolates SnB (**A**), Sn9MN-3A (**B**) and Sn4VD (**C**) during 5 days (*1*) and 14 days (*2*, *3*) of cultivation in PGA medium and on sporulation after 14 days of growth (**D**). The variants in the table marked with similar letters do not differ significantly according to Duncan’s test (*n* = 9, *p* ≤ 0.05).

The colony area of the Sn4VD isolate on the 5th day of cultivation was 8 cm^2^, while the area of mycelium of the two virulent isolates SnB and Sn9MN-3A was approximately the same and 28–34% less than that of the avirulent isolate Sn4VD (Figure 2B). On the 14th day of cultivation, the trend of the mycelial growth remained unchanged; the mycelial area of avirulent isolate Sn4VD exceeded the mycelial area of two virulent isolates by 17–24% (Figure 2B). The influence of zeatin and ABA on the radial growth of SnB and Sn9MN-3A isolates was approximately the same. Both PGRs inhibited the mycelial growth of these isolates to the same extent (Figure 2B). In contrast to the virulent isolates SnB and Sn9MN-3A, the avirulent isolate Sn4VD increased its radial growth in the presence of both zeatin and ABA (Figure 2B). The influence of zeatin increased the colony area of Sn4VD isolates by 48% on the 5th day of cultivation compared to the untreated control (Figure 2B). ABA increased the area of the mycelium of the Sn4VD isolate by 37% on the 5th day of cultivation compared to the untreated control (Figure 2B). On the 14th day of cultivation on the medium with or without the addition of zeatin and ABA, almost all colonies of the avirulent isolate Sn4VD reached the edge of the Petri dishes and did not differ in size (Figure 2B and Figure 3C).

Spores were found only in the two virulent isolates, SnB and Sn9MN-3A; the Sn4VD isolate did not form spores (Figure 3A–C). Isolates SnB and Sn9MN-3A formed a thin layer of mycelium with a large number of spores (Figure 3A,B). The Sn4VD isolate differed from other isolates as it had a very thick mycelial layer without spores (Figure 3C). When isolates SnB and Sn9MN-3A were cultivated under the same conditions, the number of spores formed on colonies of Sn9MN-3A isolate was two times greater than that on colonies of the SnB isolate (Figure 3D). Subsequently, we studied the effect of ABA and zeatin on the sporulation of different isolates of *S. nodorum* (Figure 3D).

The addition of zeatin to the cultivation medium increased the sporulation of the Sn9MN-3A isolate by six times, as well as of the SnB isolate (Figure 3D). The sporulation of isolate SnB was influenced to a greater extent by the addition of ABA than zeatin to the culture medium (Figure 3D). The effect of ABA on the sporulation of SnB colonies led to an eight-fold increase in this parameter when comparing non-treated colonies with PGR ones (Figure 3D). In the case of the Sn9MN-3A isolate, zeatin possessed more influence than ABA. The addition of ABA to the culture medium increased the sporulation of the Sn9MN-3A isolate by only four times (Figure 3D). The addition of ABA or zeatin to the cultivation medium of the avirulent isolate Sn4VD did not induce sporulation (Figure 3D).

#### 3.2.3. The Role of Carbohydrate Metabolism Enzymes in Sporulation of *S. nodorum* SnB

**Figure 4:**
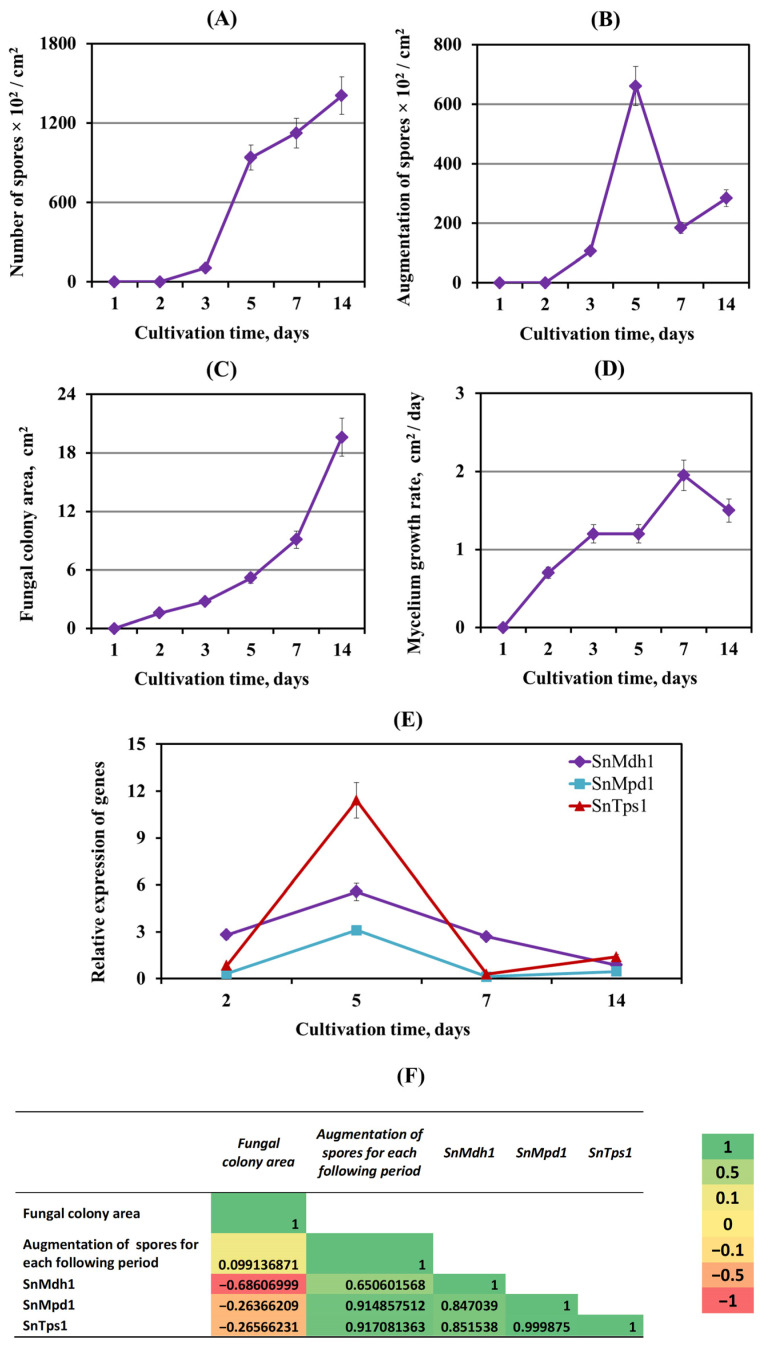
Correlation of growth, sporulation and expression of genes of carbohydrate metabolism of *S. nodorum* SnB isolate. (**A**) Number of spores; (**B**) augmentation of spores for each following period; (**C**) fungal colony area; (**D**) mycelium growth rate; (**E**) expression of the *SnMdh1*, *SnMpd1* and *SnTps*1 genes during 14 days of cultivation in PGA medium; expression values were normalized to the housekeeping fungal genes *Snβ-tubulin* and *SnEF-1α* as an internal reference; (**F**) correlation matrix representing the correlation between parameters presented in this figure. Figures present means ± SE (*n* = 9).

A comparative analysis of the growth and sporulation of the SnB isolate showed that the rapid growth of the mycelium on days 2, 3 and 7 of cultivation corresponded to a low rate of spore formation (Figure 4A,B,D,E). On the contrary, when the fungus actively sporulated (many spores were formed) on the 5th and 14th days of cultivation, the growth rate of the fungal mycelium decreased (Figure 4A,B,D,E). Correlation analysis revealed a negative relationship between mycelial growth and sporulation (Figure 4F).

Transcription analysis of the genes of carbohydrate metabolism showed that the levels of transcripts of the *SnMdh1*, *SnMpd1* and *SnTps1* genes increased on the 5th day of cultivation (active sporulation) by 2, 10.3 and 13.7 times, respectively, compared with the 2nd day of cultivation (Figure 4C).

On the 7th day of cultivation, a decrease in spore formation and acceleration of mycelial growth were observed, and the transcript levels of all genes under investigation returned to the initial values (Figure 4C). On the 14th day of cultivation, the transcript levels of genes *SnTps1* and *SnMpd1* were again slightly increased compared to the 2nd day of cultivation, which corresponds to an increase in the number of spores and a slowdown in mycelial growth (Figure 4C). Correlation analysis showed a strong positive correlation between the sporulation and the expression of carbohydrate metabolism genes *SnTps1*, *SnMdh1* and *SnMpd1* and a negative correlation between the expression of these genes and the rate of mycelial growth (Figure 4F).

#### 3.2.4. The Influence of Zeatin and ABA on the Expression of Genes Encoding Enzymes of Carbohydrate Metabolism

**Figure 5:**
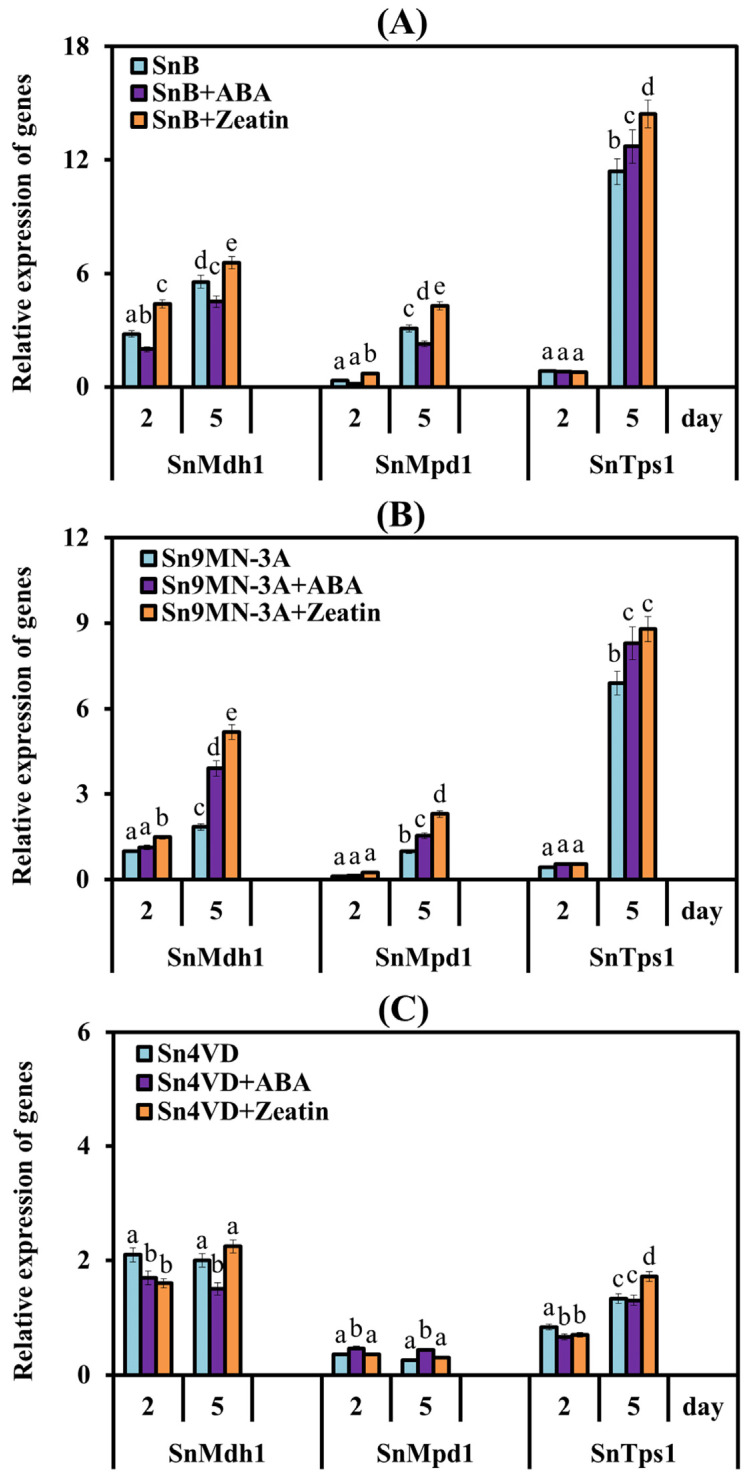
Effect of ABA and zeatin on the expression of the *SnMdh1*, *SnMpd1* and *SnTps1* genes in *S. nodorum* isolates SnB (**A**), Sn9MN-3A (**B**) and Sn4VD (**C**) after 2 and 5 days of cultivation. Expression values were normalized to the housekeeping fungal genes *Snβ-tubulin* and *SnEF-1α* as an internal reference. Figures present means ± SE (*n* = 9). The variants in histograms for each gene marked with similar letters do not differ significantly according to Duncan’s test (*p* ≤ 0.05).

The study of the expression of genes for the synthesis of trehalose and mannitol of *S. nodorum* Sn9MN-3A and Sn4VD was carried out on the 2nd and 5th days of cultivation (Figure 5B,C).

The pattern of changes in the expression of the *SnMdh1*, *SnMpd1* and *SnTps1* genes in the virulent isolate Sn9MN-3A was the same as in the SnB isolate; an increase in the levels of transcripts of all three genes was found on the 5th day of cultivation by 1.8-, 8.9- and 16-fold compared to values which were observed on the 2nd day of cultivation (Figure 5B). In the avirulent isolate Sn4VD, the levels of transcripts of the mannitol synthesis genes *SnMdh1* and *SnMpd1* were not changed, but mRNA content of the *SnTps1* gene increased on the 5th day of cultivation by 1.6 times compared to the 2nd-day values (Figure 5C).

Analysis of the influence of PGRs on the transcription of the *SnMdh1, SnMpd1* and *SnTps1* genes showed that zeatin and ABA increased the levels of transcripts of all genes under investigation in the aggressive isolates SnB and Sn9MN-3A on the 5th day of cultivation (during active sporulation) and did not affect the expression of these genes in the avirulent non-sporulating isolate Sn4VD compared with the expression level in the absence of PGRs (Figure 5).

### 3.3. The Influence of Zeatin and ABA on the Expression of Genes Encoding NEs and TFs of S. nodorum Isolates

**Figure 6:**
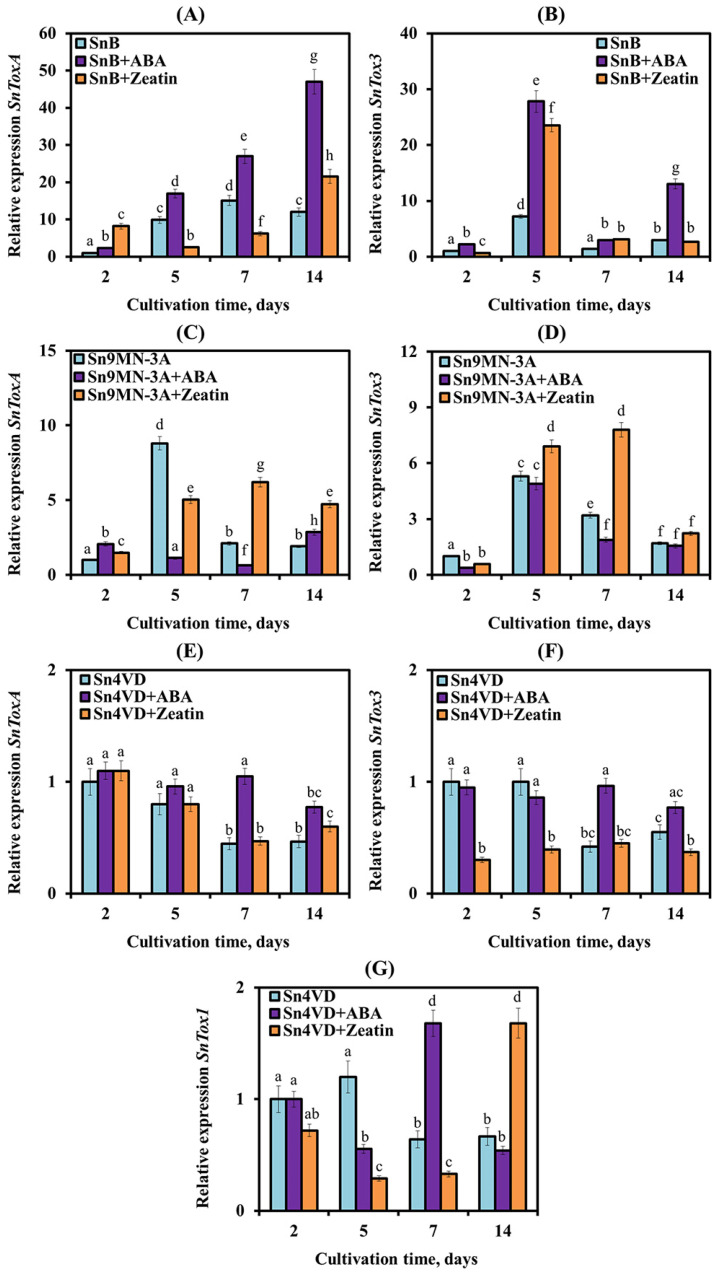
Effect of ABA and zeatin on the expression of NE genes *SnToxA* (**A**,**C**,**E**), *SnTox3* (**B**,**D**,**F**) and *SnTox1* (**G**) in *S. nodorum* isolates SnB (**A**,**B**), Sn9MN-3A (**C**,**D**) and Sn4VD (**E**,**F**,**G**) after 2, 5, 7 and 14 days of cultivation. Expression values were normalized to the housekeeping fungal genes *Snβ-tubulin* and *SnEF-1α* as an internal reference. Figures present means ± SE (*n* = 9). The variants in histograms for each gene marked with similar letters do not differ significantly according to Duncan’s test (*p* ≤ 0.05).

The expression of the *SnToxA* gene was studied in SnB, Sn4VD and Sn9MN-3A isolates of the pathogen (Figure 6A,C,E). In two virulence isolates SnB and Sn9MN-3A, an approximately 9–10-fold increase in *SnToxA* gene expression was found during active sporulation (on the 5th day of cultivation) as compared to this parameter before sporulation (the 2nd day of cultivation) (Figure 6A,C).

Subsequently, the level of *SnToxA* gene transcripts increased 15 times in the SnB isolate and only two times in the Sn9MN-3A isolate (Figure 6A,C). In the avirulent isolate Sn4VD, the expression of the *SnToxA* gene did not increase on the 5th day, and on the 7th and the 14th days of cultivation, it even decreased, while the isolate did not sporulate (Figure 6E). The greatest increase in transcript levels of the *SnTox3* gene in two of the three pathogen isolates (SnB and Sn9MN-3A) was detected during active sporulation (the 5th day of cultivation) (Figure 6B,D). Thus, the mRNA content of the *SnTox3* gene increased seven times in the SnB isolate and five times in the Sn9MN-3A isolate (Figure 6B,D). And in the avirulent isolate Sn4VD, the transcript level of the *SnTox3* gene did not change at the beginning of cultivation, and then, on the 7th and 14th days of growth, the mRNA content of the gene decreased (Figure 6F). Thus, in SnB, Sn4VD and Sn9MN-3A isolates, the expression patterns of *SnToxA* and *SnTox3* genes were different, which may be associated with different aggressiveness of these isolates. Expression of the *SnTox1* gene was studied in the Sn4VD isolate (Figure 6G). The avirulent isolate Sn4VD showed a decrease in the expression of the *SnTox1* gene during in vitro cultivation (Figure 6G).

We studied the influence of exogenous ABA and zeatin on the expression of genes encoding NEs, which claim to be regulators of the development and virulence of pathogenic fungi (Figure 6) [5,8]. The addition of ABA to the cultivation medium induced a strong increase in the expression of *SnToxA* and *SnTox3* genes in the SnB isolate (Figure 6). In the SnB isolate, ABA permanently increased the transcript level of the *SnToxA* gene by 2–4 times, with the maximum on the 14th day of cultivation, compared to the expression level in the absence of ABA (Figure 6A). The mRNA content of the *SnTox3* gene in the SnB isolate was increased under the influence of ABA four and seven times on the 5th and 14th days of cultivation, respectively, compared with the expression level in the absence of the hormone (Figure 6B). In the avirulent isolate Sn4VD, the addition of ABA induced a slight increase in the transcript level of the *SnTox3*, *SnToxA* and *SnTox1* genes on the 7th, 7th and 14th and 7th days of mycelial growth, respectively, compared to the expression level in the absence of ABA (Figure 6). In the Sn9MN-3A isolate, the addition of ABA either had no effect or slightly decreased the transcript level of the *SnToxA* and *SnTox3* genes (Figure 6C,D).

In the SnB isolate, the addition of zeatin to the culture medium induced an increase in the expression of the *SnToxA* gene by 8 and 1.8 times, respectively, on the 2nd and 14th days of cultivation compared to the expression level in the absence of zeatin (Figure 6A). The transcript level of the *SnTox3* gene with the addition of zeatin increased 4.5 times on the 5th day of cultivation compared to the expression level in the absence of zeatin, and in the remaining periods, zeatin either had a weak effect or decreased the expression of this gene (Figure 6B). On the contrary, the addition of zeatin to the culture medium of the Sn9MN-3A isolate increased the transcript level of the *SnToxA* and *SnTox3* genes during different periods of mycelial growth compared to the expression level in the absence of zeatin (Figure 6C,D). In the avirulent isolate Sn4VD, the addition of zeatin did not affect the expression of the *SnToxA* gene, reduced the transcript level of the *SnTox3* gene on the 2nd and 5th days of cultivation and increased the transcript level of the *SnTox1* gene on the 14th day of cultivation compared to the expression level in the absence of zeatin (Figure 6E–G).

**Figure 7:**
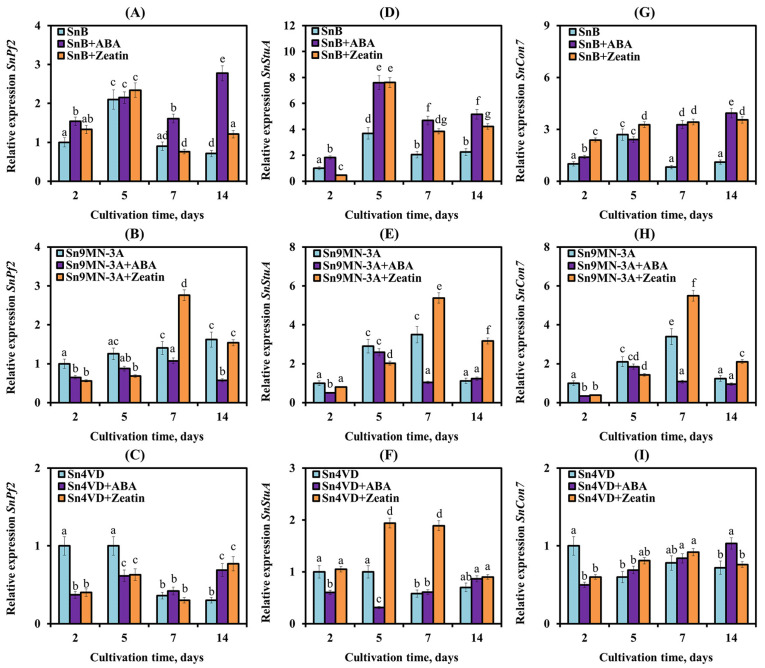
Effect of ABA and zeatin on the expression of TF genes *SnPf2* (**A**,**B**,**C**), *SnStuA* (**D**,**E**,**F**) and *SnCon7* (**G**,**H**,**I**) in *S. nodorum* isolates SnB (**A**,**D**,**G**), Sn9MN-3A (**B**,**E**,**H**) and Sn4VD (**C**,**F**,**I**) after 2, 5, 7 and 14 days of cultivation. Expression values were normalized to the housekeeping fungal genes *Snβ-tubulin* and *SnEF-1α* as an internal reference. Figures present means ± SE (*n* = 9). The variants in histograms for each gene marked with similar letters do not differ significantly according to Duncan’s test (*p* ≤ 0.05).

In our work, the expression of TF genes *SnPf2*, *SnStuA* and *SnCon7* had different patterns in the SnB, Sn4VD and Sn9MN-3A isolates (Figure 7).

In the SnB isolate, the transcript level of the *SnPf2*, *SnStuA* and *SnCon7* genes increased on the 5th day of cultivation by 2-, 3.7- and 2.7-fold, respectively, compared to the initial expression level (on the 2nd day of cultivation) (Figure 7A,D,G). In the Sn9MN-3A isolate, an increase in the mRNA content of the *SnPf2* gene by 1.6 times compared with the initial expression level was detected only on the 14th day of cultivation (Figure 7B). The transcript level of the *SnStuA* and *SnCon7* genes in the Sn9MN-3A isolate increased approximately 2–3.5 times on the 5th and 7th days of cultivation compared to the initial expression level (Figure 7E,H). In the Sn4VD isolate, a decrease in the transcript level of *SnPf2* and *SnStuA* genes on the 7th and 14th days of cultivation compared to the initial expression level was observed (Figure 7C,F). Expression of the *SnCon7* gene decreased from the 5th day of cultivation in the avirulent isolate Sn4VD (Figure 7I).

The addition of ABA and zeatin to the cultivation medium affected the expression of TF genes in various isolates in the same way as in the expression of NE genes (Figure 7). ABA had the greatest effect on the expression of TF genes in the SnB isolate. ABA increased the transcript level of *SnStuA*, *SnPf2* and *SnCon7* genes on the 7th and 14th days of cultivation of the SnB isolate, when in the absence of ABA, the expression of these genes had already decreased (Figure 7A,D,G). In the Sn4VD and Sn9MN-3A isolates, the addition of ABA had either no or little effect on the expression of TF genes and mainly led to a decrease in the transcript level of *SnStuA*, *SnPf2* and *SnCon7* genes in the Sn9MN-3A isolate on the 7th day of cultivation (Figure 7).

On the contrary, the addition of zeatin to the cultivation medium increased the transcript level of the *SnStuA*, *SnPf2* and *SnCon7* genes in the Sn9MN-3A isolate and did not affect the expression of the *SnPf2* gene in the SnB isolate but increased the transcript level of the *SnStuA* and *SnCon7* genes (Figure 7). In the Sn4VD isolate, the addition of zeatin did not affect the mRNA content of the *SnCon7* gene but mainly increased the transcript level of the *SnStuA* gene and mainly decreased the mRNA content of the *SnPf2* gene compared with the expression level in the absence of the hormone (Figure 7).

**Figure 8:**
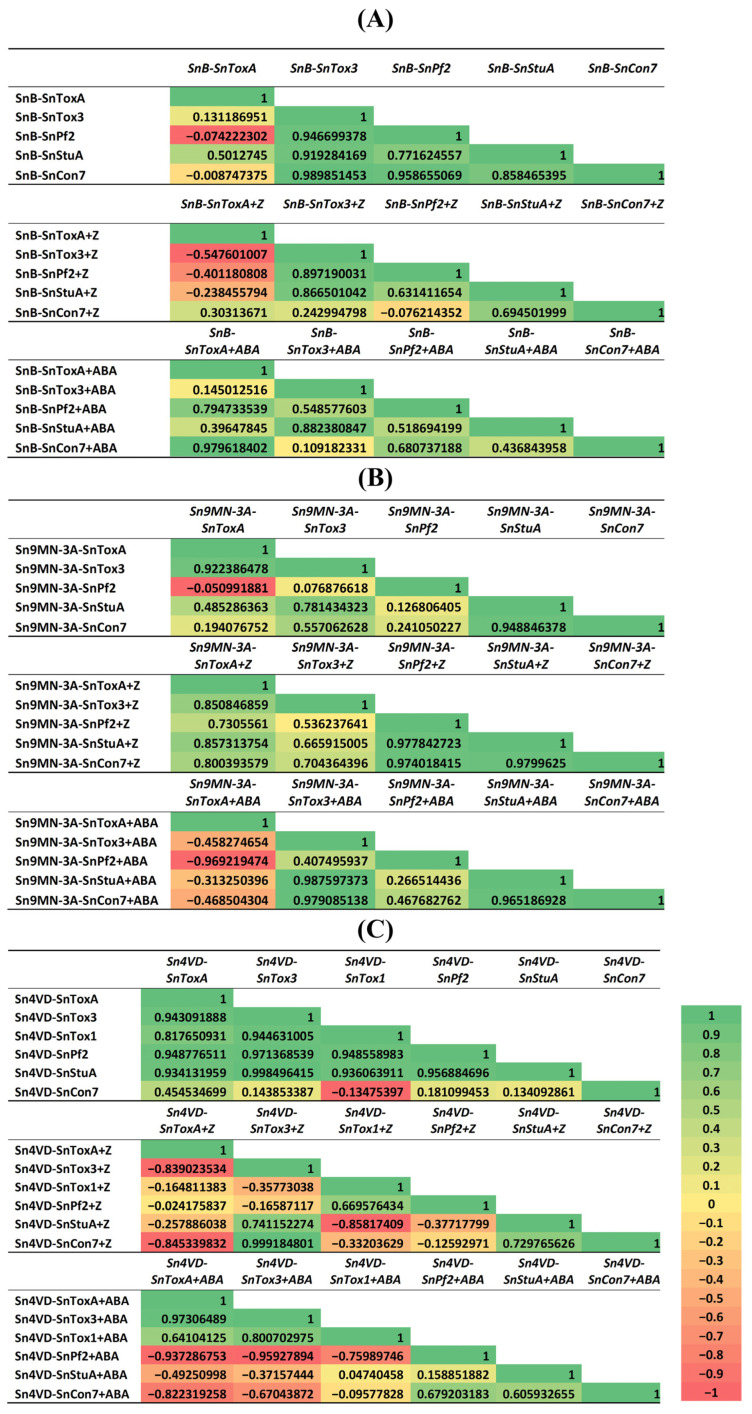
Correlation matrix representing the correlation between the expression of NE genes and TF genes in *S. nodorum* isolates SnB (**A**), Sn9MN-3A (**B**) and Sn4VD (**C**).

Correlation analysis showed that in the SnB isolate, the expression of the *SnPf2*, *SnStuA* and *SnCon7* genes was strongly correlated with the expression of the *SnTox3* gene. The expression of the *SnToxA* gene was weakly correlated only with the expression of the *SnStuA* gene (Figure 8A). In the Sn9MN-3A isolate, the expression parameters of NE and TF genes had a weaker correlation with each other (Figure 8B). Thus, the correlation between the expression of the *SnTox3* and *SnPf2* genes was weaker, and the correlation between the expression of the *SnToxA* and *SnStuA* genes was stronger than that of the parameters of the SnB isolate (Figure 8). In the Sn4VD isolate, the expression of the NE and TF genes demonstrated a high correlation with each other (Figure 8C).

Zeatin discontinued the correlation between NE and TF gene expression in SnB and Sn4VD isolates (Figure 8). Thus, in the SnB isolate, the addition of zeatin to the culture medium weakened the correlation between the expression of the *SnToxA* gene and the *SnStuA, SnPf2* genes, *SnTox3* and *SnCon7* genes (Figure 8A). Zeatin weakened the connections between the expression of the *SnToxA* and *SnTox3* genes and all TFs in the Sn4VD isolate, and the correlation between the expression of the encoding *SnTox3* and *SnPf2* genes was especially weakened (Figure 8C). However, the addition of zeatin to the culture medium of the Sn4VD isolate enhanced the positive correlation between the expression of *SnTox3* and *SnCon7* genes (Figure 8C). Also, zeatin enhanced the correlation between the expression of *SnToxA* and *SnTox3* genes with all TFs, especially between the expression of the *SnTox3* gene with the *SnPf2* and *SnCon7* genes and the *SnToxA* gene with the *SnPf2* gene in the Sn9MN-3A isolate (Figure 8B).

The addition of ABA increased the correlation between the expression of NE and TF genes in virulent isolates SnB and Sn9MN-3A (Figure 8A,B). Thus, in the SnB isolate, ABA increased the positive correlation between the expression of the *SnToxA* gene and all TF genes (Figure 8A), weakened the relationship between the expression of the *SnTox3* gene and the *SnPf2* and *SnCon7* genes and did not affect the correlation between the expression of the *SnTox3* gene and the *SnStuA* genes (Figure 8A).

In the Sn9MN-3A isolate, ABA increased the correlation between the expression of the *SnTox3* gene and all TF genes (Figure 8B) but weakened the relationship between the expression of the *SnToxA* gene with all the TF genes (Figure 8C). In the Sn4VD isolate, ABA disrupted all correlations between the expression of genes encoding NEs and TFs (Figure 8C).

Thus, correlation analysis showed that zeatin and ABA could influence the virulence of isolates, but their influence depended on the genotype of the isolate.

## 4. Discussion

### 4.1. Why Do Fungi Need Hormones?

It is well known that fungi can produce compounds similar to hormones [5,8]. Fungal hormones can influence the physiology of the fungi themselves and disrupt various processes in plants, promoting invasion and absorption of nutrients by pathogens [5,8]. The results of this work showed that all *S. nodorum* isolates synthesized ABA and IAA in vitro, but only Sn9MN-3A and Sn4VD synthesized CKs in sufficient quantities. Also, trace amounts of CKs were detected in isolate SnB (Table 1). Therefore, the balance of synthesized hormones in the aggressive isolates SnB and Sn9MN-3A was shifted towards ABA and IAA, and in the avirulent isolate Sn4VD, the hormonal balance was shifted towards CKs and IAA (Table 1).

Many species of fungi, bacteria and other microorganisms produce and secrete auxins [5]. It is reliably known that auxins promote the growth of fungal hyphae, the germination of spores and the sporulation rate of fungi [5]. On the contrary, CKs are synthesized mainly by (hemi)biotrophic fungal pathogens and some necrotrophic fungi with a small biotrophic phase in their lifestyle [8,39,40]. The function of these CKs is currently unclear [8]. However, it has been suggested that CKs play an important role in the development of hyphae, fungal virulence and nutrient uptake since it has been experimentally proven that many fungi can sense CKs [5,8,10,11]. ABA is synthesized by many fungi with different lifestyles (saprophytic, symbiotic and pathogenic) [5,14,15,17]. Pathogenic fungi use ABA most often as a virulence factor, which can increase plant susceptibility and accelerate the aggressiveness of the fungus itself [14,41]. Unfortunately, there are very limited studies on the effect of ABA on the growth and development of fungi [15,17].

In this work, we investigated the role of exogenous CKs (zeatin) and ABA on the growth, sporulation and virulence factors of the fungus *S. nodorum*. In addition, the tested isolates themselves produced CKs and ABA or ABA individually (Table 1). We used a concentration of zeatin and ABA of 0.1 μM, which is physiological for the studied *S. nodorum* isolates (Table 1). The addition of ABA to the cultivation medium of virulent isolates SnB and Sn9MN-3A increased the total level of the hormone by 1.5–2 times. The addition of zeatin to the culture medium increased the hormone content in Sn9MN-3A by three times and in SnB by about 30 times, since trace amounts of CKs were detected in this isolate (Table 1). The addition of PGRs to the cultivation medium of the avirulent isolate Sn4VD increased the level of ABA by three times and CKs by only 20%, since this isolate produced quite a lot of this hormone (Table 1). Thus, in our work, we used three isolates of the pathogen *S. nodorum* with different genotypes, approximately the same level of ABA synthesis and different levels of CK synthesis.

### 4.2. The Influence of CKs and ABA on the Growth and Sporulation of S. nodorum

Our results showed that virulent isolates SnB and Sn9MN-3A grew and sporulated quite well in vitro on PGA, but the Sn9MN-3A isolate produced two times more spores than the SnB isolate (Figure 2 and Figure 3). The avirulent isolate Sn4VD grew faster than virulent isolates but did not form spores on PGA (Figure 2 and Figure 3). The addition of zeatin or ABA to the cultivation medium inhibited growth and increased the sporulation of virulent isolates SnB and Sn9MN-3A by 4–8 times and did not induce sporulation of the avirulent isolate Sn4VD or inhibit its growth (Figure 2 and Figure 3). It is worth noting that zeatin had an equally strong effect on the sporulation of both isolates, and ABA had a stronger effect on the sporulation of the SnB isolate (Figure 3).

The role of CKs in the direct inhibition of the growth of fungal phytopathogens was recently demonstrated [10]. Various concentrations of a CK (zeatin) and its analogs (6-benzylaminopurine (6-BAP), kinetin, adenine and bacterial non-cyclic thidiazuron (TDZ)) inhibited the growth of the mycelium of various pathogens of tomato, such as *Botrytis cinerea*, *Sclerotium rolfsii* and *Fusarium oxysporum*, by 20–60% compared to colonies untreated with hormones [10]. There are no data on the effect of exogenous ABA on the growth of fungal mycelium. Studies with mutant strains of *B. cinerea* with altered ABA synthesis showed that ABA is necessary for normal fungal growth, but its effect depended on the concentration of the hormone [15,17]. Increased ABA synthesis led to slower growth of mutant strains [15].

Information on the direct effect of CKs and ABA on the sporulation of ascomycetes is limited; this issue has practically not been studied yet. Back in the 1960s, the effect of CKs on reproduction in ascomycetes was reported [42]. However, there was one study that reported that 0.1 μM 6-BAP decreased sporulation of *B. cinerea* in vitro by 50% compared to the control ones [10]. The direct effect of ABA on the sporulation of ascomycetes was addressed in one study, which showed that exogenous ABA increased spore germination and appressorium formation of the necrotrophic fungus *Magnaporthe oryzae* [14]. Thus, the literature is insufficient, and further study of this issue is necessary.

What could be the mechanism of action of CKs and ABA on the growth and sporulation of fungi? We hypothesize that the mechanism of action of CKs and ABA is associated with the regulation of carbohydrate metabolism. Recently, some studies have shown that the CKs and ABA play an important role in the regulation of carbohydrate metabolism in ascomycetes [11,15,17].

Asexual sporulation in filamentous fungi of the Ascomycota division, which includes the pathogen *S. nodorum*, requires the regulation of gene expression, specialized cell differentiation and response to environmental factors, as studied in the model saprophytic fungus *Aspergillus nidulans* [43]. Previous studies of *S. nodorum* have identified several genes and metabolic pathways involved in asexual sporulation [44,45]. Screening for sporulation-related metabolites in *S. nodorum* showed that trehalose, a disaccharide carbohydrate containing two D-glucose residues, accumulated significantly during asexual sporulation both in vitro and in planta [44]. Using the *S. nodorum* mutant deficient in trehalose synthesis, with a deletion in the *Tps1* gene encoding trehalose-6-phosphate synthase, the role of trehalose in the formation and germination of pycnidiospores was proven [44].

It has also been shown that sporulation of *S. nodorum* requires mannitol, just like the fungus *Beauveria bassiana* [45,46]. Mannitol is an acyclic hexitol, which is found in most higher fungi and acts as a carbohydrate storage site [45]. In the work of P. S. Solomon et al. [45], double mutants of *S. nodorum* were obtained for the genes encoding enzymes of mannitol biosynthesis, mannitol 2-dehydrogenase (gene *Mdh1*), which directly reduces fructose to mannitol, and mannitol-1-phosphate dehydrogenase (gene *Mpd1*), which reduces fructose-6-phosphate to mannitol-1-phosphate [45]. These double-mutant strains were unable to sporulate in vitro when grown on a minimal medium for extended periods. Deficiency in sporulation was correlated with the depletion of intracellular mannitol pools [45].

Our results showed that the sporulation of virulent isolates SnB and Sn9MN-3A strictly correlated with the induction of the expression of the carbohydrate metabolism genes *SnTps1*, *SnMdh1* and *SnMpd1* (Figure 4 and Figure 5). Our results demonstrated that zeatin and ABA increased the levels of transcripts of the *SnTps1*, *SnMdh1* and *SnMpd1* genes in isolates SnB and Sn9MN-3A (Figure 5), which correlated with an increase in sporulation of the SnB and Sn9MN-3A colonies (Figure 3). It is worth noting that the addition of zeatin to the cultivation medium affected both the expression of carbohydrate metabolism genes and sporulation to a greater extent than ABA (Figure 3 and Figure 5).

Thus, our results revealed the regulatory role of CKs and ABA in the processes of growth, development and asexual reproduction of the fungal pathogen *S. nodorum*. This work shows that the influence of hormones on these processes can be opposite and depends both on the concentration of hormones and on the genotype of the pathogen. We assume that the influence of hormones on the growth and sporulation of the pathogen *S. nodorum* is carried out through the regulation of carbohydrate metabolism.

### 4.3. The Influence of CKs and ABA on the Virulence Factors of the Fungus S. nodorum

Another vector of action of fungal-derived hormones is the virulence of the pathogen. The pathogen *S. nodorum* uses NEs as virulence factors on wheat varieties carrying the corresponding dominant susceptibility genes [20]. This fact has been well proven using *S. nodorum* mutants carrying deletions in the effector genes, which experienced a loss of virulence [30]. It was previously shown that the virulence of *S. nodorum* strains depends on the level of expression of NE genes [26,27,30,47,48]. Isolates SnB, Sn4VD and Sn9MN-3A studied in this work had different virulence [32,33], and as our results showed, they differed in the pattern of expression of NE genes (Figure 6). Isolates SnB and Sn9MN-3A were characterized by higher expression of the *SnToxA* and *SnTox3* genes compared to the avirulent isolate Sn4VD (Figure 6). Also, low expression of the *SnTox1* gene was detected in the Sn4VD isolate (Figure 6), which corresponds to the results previously obtained by other authors. Thus, two early studies showed that higher expression of the NE gene *SnToxA* led to increased rates of disease in wheat [47,49]. The work of other researchers has shown that *S. nodorum* SN15 with lower expression of the *SnTox3* gene was less aggressive, but the addition of the SnTox3 protein to its filtrate increased its virulence [26]. Expression of the *SnTox1* gene also varied among *S. nodorum* isolates [27]. It was higher in isolates SN2000 and SN15 than in isolates SN4 and SN6, and these rates correlated with stronger disease progression mediated by the SnTox1–*Snn1* interaction [23,48,50].

The exact mechanism of differential expression of NE genes among *S. nodorum* strains remains unclear, but based on recent discoveries, this phenomenon can be explained by the presence of genetic polymorphisms in the promoter region of NE genes containing regulatory elements that are targets for specific proteins [27,30]. Recently, the importance of some TFs as regulatory elements targeting key pathogen virulence pathways has been established [28]. In our work, the expression of TF genes *SnPf2*, *SnStuA* and *SnCon7* was studied (Figure 7). Our results showed that the expression of TF genes correlated with the expression of NE genes (Figure 8), which suggests their regulatory role and coincides with the literature data [28]. The correlation analysis carried out in this work showed that in virulent isolates SnB and Sn9MN-3A, the expression of the *SnTox3* gene correlated with the expression of TF genes *SnPf2*, *SnStuA* and *SnCon7*, and the expression of the *SnToxA* gene weakly correlated with the expression of the *SnStuA* gene (Figure 7 and Figure 8), which coincides with the literature data [29,30]. Earlier, functional analysis showed that *SnPf2* acts as a positive regulator of the expression of genes encoding *SnToxA* and *SnTox3* but not *SnTox1* [30]. Deletion of the *SnPf2* gene in *S. nodorum* led to the suppression of the expression of genes encoding *SnToxA* and *SnTox3*, which led to the loss of its host-specific virulence in wheat [30]. Studies of NE gene expression in *S. nodorum* have shown that *SnStuA* is a positive regulator of *SnTox3*, but it is not required for *SnToxA* expression [29]. It was also previously proven that *SnCon7* directly connects to the promoter region of the *SnTox3* gene [31]. TF SnCon7 also regulates the expression of two other NE genes, *SnTox1* and *SnToxA*, but this regulation is indirect [31].

Along with TFs, the virulence of pathogens can be regulated by hormones of fungal origin [5]. However, the role of hormones in the regulation of the expression of NE and TF genes has practically not been studied. Our results showed that ABA and zeatin influenced the expression of NE genes *SnToxA*, *SnTox3* and *SnTox1* and TF genes *SnPf2*, *SnStuA* and *SnCon7* in all isolates, SnB, Sn9MN-3A and Sn4VD (Figure 6 and Figure 7).

The addition of ABA to the cultivation medium of the SnB isolate increased the expression of two NE genes and all three TF genes (Figure 6 and Figure 7). Despite such a strong positive effect of ABA on the expression of NE and TF genes, correlation analysis showed that ABA could increase the virulence of the SnB isolate associated with the expression of the *SnToxA* gene and reduce the virulence associated with the expression of the *SnTox3* gene (Figure 8). ABA had less effect on Sn9MN and Sn4VD isolates than on the SnB isolate (Figure 6 and Figure 7). However, correlation analysis showed that in the Sn9MN-3A isolate, ABA could increase virulence associated with the expression of the *SnTox3* gene and reduce virulence associated with the expression of the *SnToxA* gene, and in the Sn4VD isolate, ABA disrupted all correlations between NEs and TFs (Figure 8). This fact may be associated with a different use of the ABA hormonal signaling pathway by the SnTox3 and SnToxA effectors since effectors are known to manipulate hormonal signaling pathways [2]. In addition, isolates SnB and Sn9MN-3A can produce other yet undiscovered NEs, which can determine the relationship of the isolate with a particular PGR. As shown by correlation analysis, the influence of ABA on the effectors of the isolates was carried out through the regulation of the expression of *SnPf2* and *SnCon7* genes (Figure 8). There is a lot of evidence that ABA often suppresses host immune responses and that PGR can be used by pathogens (*Fusarium oxysporum*, *Phytophthora infestans, Cladosporium cucumerinum*, *Magnaporthe grisea* and *M. oryzae*) as an effector molecule [14,41,51,52,53]. However, only one study proved the role of this hormone in the virulence of *M. oryzae* using a knockout mutant impaired in ABA biosynthesis [14]. ABA is also considered as a communication signal between different species during the development of both mutualistic host–microbe interactions and host–pathogen relationships [15,41]. Our results show that the effect of ABA on the virulence factors of the pathogen *S. nodorum* could depend on both the genotype of the isolate (a set of different effectors) and the concentration of the hormone (Table 1), which coincides with the literature data [5,15,41].

The addition of a CK (zeatin) to the culture medium of the Sn9MN-3A isolate increased the expression of two NE genes and all three TF genes (Figure 6 and Figure 7). As the correlation analysis showed, zeatin could increase the virulence of the Sn9MN-3A isolate associated with NEs SnToxA and SnTox3 (Figure 8). Despite the fact that the addition of zeatin to the culture medium of isolates SnB and Sn4VD increased the expression of some NE and TF genes (Figure 6 and Figure 7), zeatin discontinued the correlation between the expression of NE and TF genes, which could lead to a decrease in the virulence of isolates (Figure 8). As shown by correlation analysis, the influence of zeatin on the effectors of the isolates under investigation was also carried out through the regulation of the expression of *SnPf2* and *SnCon7* genes, as in the case of ABA (Figure 8). Little is known about the role of CKs in the virulence of fungal pathogens, but most of these studies show that CKs are responsible for the virulence of biotrophic pathogens that form tumors in their hosts, such as *U. maydis* [5,54]. However, it has also been shown that CKs are required for full virulence of the hemibiotrophic non-tumor-forming pathogen *M. oryzae* [55]. On the other hand, a recent study of the *B. cinerea* transcriptome using RNA next-generation sequencing showed that the addition of CKs to the cultivation medium suppressed the virulence of the fungus due to the influence on the genes of virulence factors [10]. Our results showed that the genotype of the isolate (a set of different effectors) and the concentration of CKs can be the determining factors in the vector of the hormone’s action, which coincides with the literature data [5,56].

It is known that in addition to virulence, many fungal TFs influence the vital processes of fungi, controlling various aspects of metabolism, development and stress resistance of fungi, just like hormones [5,8,28]. Thus, TF SnPf2 regulates carbohydrate metabolism, resistance to abiotic stress factors, infectious morphogenesis and sporulation [28]. TF SnStuA is responsible for sporulation, carbohydrate metabolism, glycolysis, the tricarboxylic acid cycle and amino acid synthesis [29]. TF SnCon7 regulates sporulation in fungi and is also an important factor for penetration into host plant cells [28]. Thus, CKs and ABA, influencing the expression of TF genes, can determine not only the virulence of the isolate but can also influence carbohydrate metabolism and sporulation of the pathogen. In other words, CKs and ABA are actively involved in the growth, development and pathogenicity of the fungus *S. nodorum*.

## 5. Conclusions

Our results showed that the fungal pathogen *S. nodorum* synthesizes the hormones ABA, IAA and CKs. Using exogenous ABA and CK (zeatin), we proved that the effect of these PGRs on the growth and sporulation of *S. nodorum* isolates is carried out through the regulation of carbohydrate metabolism and depends on both the genotype of the isolate and the concentration of the hormone. ABA and CKs are able to regulate the expression of the fungal TF and NE genes. Based on our results and literature data analysis, it can be assumed that the CKs and ABA influence virulence factors (NEs) indirectly through control of the growth processes, metabolism and expression of TF genes. The fine point of hormonal regulation of fungal TFs requires further in-depth study, in which it is worth paying attention to the regulation of carbohydrate metabolism by both hormones and TFs.

## Figures and Tables

**Table 1:** Content of hormones in fungal mycelium and culture medium of various isolates of *S. nodorum*.

Parameter	Isolate *S. nodorum*	ABA	IAA	Cytokinins *
Hormone Level, ng/g Fresh Weight in Fungal Mycelium	SnB	36.9 ± 2.8 a	79.8 ± 1.9 b	0.7 ± 0.01 d
	Sn9MN-3A	21.9 ± 1.6 b	76.2 ± 3.9 b	12.2 ± 1.2 b
	Sn4VD	13.9 ± 0.4 c	143.4 ± 15.0 a	121.9 ± 9.3 a
Hormone Level, ng/mL of Culture Medium	SnB	15.7 ± 1.0 c	20.5 ± 1.2 d	0.05 ± 0.001 d
	Sn9MN-3A	12.1 ± 0.8 c	20.6 ± 3.5 d	3.0 ± 0.6 c
	Sn4VD	17.7 ± 1.7 bc	42.9 ± 4.1 c	10.7 ± 0.9 b
	CM **	0.83 ± 0.01 d	0.08 ± 0.001 e	0.1 ± 0.005 d

* Cytokinins, the sum of zeatin and zeatin riboside. IAA—indoleacetic acid, ABA—abscisic acid. ** CM—culture medium. The variants in the same column marked with similar letters do not differ significantly according to Duncan’s test (*n* = 9, *p* ≤ 0.05).

## Data Availability

Data are contained within the article and Supplementary Files.

## Supplementary Materials

The following supporting information can be downloaded at: https://www.mdpi.com/article/10.3390/biom14050517/s1, Table S1: Primers used for real-time PCR.
