# Supplementary material for: The Role of Cytokinins and Abscisic Acid in the Growth, Development and Virulence of the Pathogenic Fungus Stagonospora nodorum (Berk.)
Source: Biomolecules. 2024 Apr 25;14(5):517. doi: 10.3390/biom14050517 (PMC11117529; doi:10.3390/biom14050517)
Supplement: Supplementary file 1 [file biomolecules-14-00517-s001.zip › biomolecules-2914019-supplementary.pdf]

Article

The Role of Cytokinins and Abscisic Acid in the Growth, Development and Virulence of the Pathogenic Fungus *Stagonospora nodorum* (Berk.)

Tatyana V. Nuzhnaya [1,2], Antonina V. Sorokan [1], Guzel F. Burkhanova [1], Igor V. Maksimov [1] and Svetlana V. Veselova [1,*]

[1] Institute of Biochemistry and Genetics, Ufa Federal Research Centre, Russian Academy of Sciences, Prospekt Oktyabrya, 71, 450054 Ufa, Russia; tanyawww89@mail.ru (T.V.N.); fourtyanns@googlemail.com (A.V.S.); guzel_mur@mail.ru (G.F.B.); maksimov@ufaras.ru (I.V.M.)

[2] Ufa Institute of Biology, Ufa Federal Research Centre, Russian Academy of Sciences, Prospekt Oktyabrya, 69, 450054 Ufa, Russia

* Correspondence: veselova75@rambler.ru; Tel.: +7-9173423941

Supplementary Table S1. Primers used for real-time PCR.

Genes	Strand	5′ to 3′ Primer Sequences	GenBank accession number
Sn_StuA	Forward	ACCACCGTCTAGCAATCTTTAC	SNOG_14941
	Reverse	GGTATCCTTGGCTCGCATATT	
Sn_Con7	Forward	CTCACGAGCTTTGACGACTT	SNOG_08362
	Reverse	CATGACCCATCCGTAAAGAGAG	
Sn_Pf2	Forward	CATTCATCAGTCTCTGGAACCG	SNOG_00649
	Reverse	CGAATCTCGACGCCTTGGG	
SnToxA	Forward	AACGCCAATACAGTGCGAGT	JX997419
	Reverse	GCTGCATTCTCCAATTTTCACG	
SnTox1	Forward	GTACTCCCGTACGTACTCTTCT	JX997402
	Reverse	CGCTTGTTTGCCGTTCTTAC	
SnTox3	Forward	CGAGCTGATATCCCGTTTGA	FJ823644
	Reverse	GGGACAGTGACAATAGGTAAGG	
Snβ-tubulin	Forward	ACACCAGGAACAACTGCTAACAGC	S56922
	Reverse	TATGCGCGCGTGCTGCAAATTCGA	
SnMpd1	Forward	CGATAGTTGGTGGTGGAATCTC	SNOG_15763
	Reverse	TGTGGTGCTGCTTCGTATATC	
SnMdh1	Forward	ACGCAGACGAACGATACATTAC	SNOG_15488
	Reverse	ACGGTAATAGCTACAGGGAAGA	
SnTps1	Forward	AATATGAGGAGGGCCGTAAAC	SNOG_03369
	Reverse	GGTCGCTCACAACGTCTATAA	
SnEF-1α	Forward	CTCATCGTCGCCATCAAC	GU456285.1
	Reverse	TGTTGTCGCCGTTGAATC	
